# Upregulated inwardly rectifying K^+^ current-mediated hypoactivity of parvalbumin interneurons underlies autism-like deficits in *Bod1*-deficient mice

**DOI:** 10.7555/JBR.38.20240394

**Published:** 2025-03-31

**Authors:** Chen Li, Kerui Wang, Xingfeng Mao, Takuya Sasaki, Xiuxiu Liu, Yingmei Lu

**Affiliations:** 1 Key Laboratory of Modern Toxicology of Ministry of Education; School of Basic Medical Sciences, Nanjing Medical University, Nanjing, Jiangsu 211166, China; 2 School of Basic Medical Sciences, Nanjing Medical University, Nanjing, Jiangsu 211166, China; 3 Department of Pharmacology, Graduate School of Pharmaceutical Sciences, Tohoku University, Sendai 980-8578, Japan; 4 Medical Basic Research Innovation Center for Cardiovascular and Cerebrovascular Diseases, Ministry of Education; International Joint Laboratory for Drug Target of Critical Illnesses; Key Laboratory of Cardiovascular & Cerebrovascular Medicine; School of Pharmacy, Nanjing Medical University, Nanjing, Jiangsu 211166, China

**Keywords:** parvalbumin neuron, biorientation defective 1, inwardly rectifying K^+^ current, autism spectrum disorder

## Abstract

Parvalbumin-positive (PV^+^) interneuron dysfunction is believed to be linked to autism spectrum disorder (ASD), a neurodevelopmental disorder characterized by social deficits and stereotypical behaviors. However, the mechanisms behind PV^+^ interneuron dysfunction remain largely unclear. Here, we found that a deficiency of Biorientation Defective 1 (*Bod1*) in PV^+^ interneurons led to an ASD-like phenotype in *Pvalb-Cre*;*Bod1*^*f/f*^ mice. Mechanistically, we observed that *Bod1* deficiency induced hypoactivity of PV^+^ interneurons and hyperactivity of calcium/calmodulin-dependent protein kinase Ⅱ alpha (CaMKⅡα) neurons in the medial prefrontal cortex, as determined by whole-cell patch-clamp recording. Additionally, *Bod1* deficiency decreased the power of high-gamma oscillation, assessed by *in vivo* multi-channel electrophysiological recording. Furthermore, we found that *Bod1* deficiency enhanced the inwardly rectifying K^+^ current, leading to an increase in the resting membrane potential of PV^+^ interneurons. Importantly, the gain-of-function of *Bod1* improved social deficits and stereotypical behaviors in *Pvalb-Cre*;*Bod1*^*f/f*^ mice. These findings provide mechanistic insights into the PV^+^ interneuron dysfunction and suggest new strategies for developing PV^+^ interneuron-targeted therapies for ASD.

## Introduction

Autism spectrum disorder (ASD), a heterogeneous neurodevelopmental disorder, is characterized by core symptoms such as social deficits and repetitive behaviors^[1]^. The global incidence of ASD is approximately 1%, with a male-to-female ratio of 4∶1^[2–3]^. Although risperidone and aripiprazole are used to manage ASD-related irritability, and atomoxetine is prescribed for ASD-related attention deficit and hyperactivity disorder, no approved drugs specifically target the core symptoms of ASD^[4–5]^. Therefore, understanding cellular mechanisms and developing pharmacological strategies to alleviate these core symptoms is crucial for ASD research.

Several studies indicate that dysfunction in the balance between neuronal excitation and inhibition is associated with ASD^[6–7]^. Parvalbumin-positive (PV^+^) interneurons, a subclass of GABAergic interneurons, play a pivotal role in regulating neuronal excitability and synchrony within neural circuits^[8]^. Growing evidence suggests that PV^+^ interneuron dysfunction significantly contributes to ASD pathogenesis^[9–10]^. Chemogenetic activation of PV^+^ interneurons in the hippocampal CA3/CA2 regions rescues social recognition impairments in *Dyrk1a*-deficient ASD mice^[11]^. Similarly, optogenetic activation of PV^+^ interneurons in the medial prefrontal cortex (mPFC) reverses social deficits in neuroligin 3 R451C knock-in mice^[12]^. Conversely, in *Htr3a* knockout mice, the inhibition of hippocampal CA1 PV^+^ interneurons ameliorates ASD-like behaviors^[9]^. These findings suggest that PV^+^ interneurons in different brain regions may play distinct roles in ASD pathology. However, the molecular mechanisms underlying PV^+^ interneuron dysfunction remain largely unexplored and warrant further investigation.

The Biorientation Defective 1 (BOD1) protein promotes chromosome biorientation and ensures proper chromosome segregation by regulating protein phosphatase 2A (PP2A) activity during mitosis^[13–14]^. Recently, BOD1 has been found at considerable levels in postmitotic brain tissues, where its dysfunction contributes to brain diseases in a mitosis-independent manner. Whole-exome sequencing from patients suggests that BOD1 dysfunction is associated with intellectual disability^[15]^. In the fruit fly *Drosophila melanogaster*, *Bod1* knockdown in postmitotic neurons induces abnormal synapse morphology and intellectual disability^[16]^. Additionally, *Bod1* deficiency in mitral/tufted cells of the olfactory bulb impairs cognitive function in *Bod1*-deficient mice by disrupting coherent activity between the olfactory bulb and cortical neural circuits^[17]^. Furthermore, BOD1 loss in cerebellar Purkinje cells reduces their excitability and spine density, leading to ataxia behaviors^[18]^. However, the role of BOD1 in PV^+^ interneurons in the context of ASD remains unclear.

In the present study, we aimed to investigate the role of BOD1 in PV^+^ interneurons in ASD. To achieve this, we generated PV^+^ interneuron-specific *Bod1*-deficient mice and used *ex vivo* whole-cell patch-clamp recordings and *in vivo* multi-channel recordings to investigate the effects of BOD1 on PV^+^ interneuron activity and neural oscillation. Additionally, we used gain-of-function approaches to determine whether BOD1 overexpression could rescue ASD-like behaviors.

## Materials and methods

### Animals

*Pvalb*-*Cre* (Strain #008069) and *Ai14* (Strain #007908) mice were obtained from the Jackson Laboratory (Bar Harbor, ME, USA). *Bod1*^*f/f*^ mice were constructed by the Nanjing Biomedical Research Institute of Nanjing University (Nanjing, Jiangsu, China) *via* homologous recombination, with loxP sites inserted upstream of the 5′ UTR and intron 2-3 of the *Bod1* gene^[18]^. To generate *Pvalb-Cre;Bod1*^*f/f*^ mice, *Pvalb*-*Cre* mice were crossed with *Bod1*^*f/f*^ mice. To control for potential effects of the *Pvalb*-*Cre* transgene itself, *Pvalb*-*Cre* mice were used as the control group in all behavioral tests.

*Pvalb-Cre* mice were crossed with *Ai14* mice to generate *Pvalb-Cre;Ai14* mice. Subsequently, *Pvalb-Cre;Bod1*^*f/f*^ mice were crossed with *Pvalb-Cre;Ai14* mice to generate *Pvalb-Cre;Bod1*^*f/f*^*;Ai14* mice. Specifically, we used *Pvalb-Cre;Ai14* mice as the control group and *Pvalb-Cre;Bod1*^*f/f*^*;Ai14* mice as the experimental group to assess the effects of *Bod1* deficiency in PV^+^ interneurons. In electrophysiological tests, both *Pvalb-Cre;Bod1*^*f/f*^*;Ai14* and *Pvalb-Cre;Ai14* mice expressed red fluorescent protein (mCherry) in PV^+^ interneurons, enabling accurate identification and targeted recording of PV^+^ interneurons.

To minimize variability due to hormonal fluctuations (particularly in females), all behavioral tests were performed using 2- to 3-month-old male mice. For electrophysiological experiments, 3- to 4-week-old male mice were used. All mice were group-housed in ventilated cages under a 12 h light/dark cycle, at a temperature of 22 ℃ to 23 ℃ and a humidity of 40% to 60%. All animal experiments were approved by the Animal Ethics and Welfare Committee of Nanjing Medical University (Approval No. 2206004).

### Behavioral analysis

Male mice were used for behavioral experiments. Data were analyzed using ANY-maze software (ANY-maze, Stoelting, IL, USA) or statistically processed using a random double-blind method. Specifically, the experimenters performing the tests and analyzing the data were unaware of the group assignments throughout the study. Animals were assigned to experimental groups either according to their genotype (without randomization) or through random allocation, as appropriate for the experimental design. There was no missing data in behavioral tests, and no imputations were performed.

The open-field test was employed to assess anxiety-like behavior and locomotor activity in a plastic cube box (50 cm × 50 cm × 45 cm)^[19]^. Before the test, mice were acclimated to the testing environment. They were then placed at the center of the box, with the designated central area located 15 cm from the perimeter. A video camera recorded their movements for 5 min. The duration spent in the central zone was used as a measure of anxiety levels, while the total distance traveled indicated locomotor mobility.

The marble burying test and self-grooming behavior test were designed to examine stereotyped behaviors^[20–21]^. In the marble burying test, mice were placed in a new box (40 cm × 40 cm × 40 cm) with a 5-cm layer of sawdust at the bottom, and 36 colorful glass marbles were arranged equidistantly in a 6 × 6 grid. After 30 min, the number of marbles buried at least two-thirds of the way in the sawdust was counted. The marble burying index was calculated as (number of marbles buried/total number of marbles) × 100%. In the self-grooming behavior test, mice were placed in a plastic cubic box (50 cm × 50 cm × 45 cm) and allowed to explore freely for 10 min. The total grooming time for each mouse during this period was recorded using a randomized, double-blind method.

The three-chamber social test was conducted using a three-chamber apparatus measuring 60 cm × 40 cm × 40 cm, as previously described^[22]^. In phase 1, the test mouse was placed in the central chamber and allowed to explore freely for 10 min. In phase 2, a strange mouse (S1) was placed in a cage in the right chamber, and the test mouse was returned to the central chamber. The mice were then permitted to explore all three chambers freely for another 10 min. In phase 3, another strange mouse (S2) was introduced into an empty cage in the left chamber, and the test mouse was allowed to explore all three chambers for 10 min. The time the test mouse spent sniffing each strange mouse and their respective cages was used as an indicator of social behavior. The social preference index was calculated as (difference between exploring S1 and empty cage (E)/total time spent exploring S1 and E) × 100%. The social novelty index was calculated as (difference between exploring S2 and S1/total time spent exploring S2 and S1) × 100%. Two *Pvalb-Cre;Bod1*^*f/f*^ mice were excluded from the three-chamber social tests because of a strong preference for one location during phase 1, which would affect the results in phases 2 and 3. This exclusion was necessary to ensure the accuracy and reliability of the experimental results.

The Y-maze test was used to assess discriminative learning, working memory, and reference memory in mice^[23]^. Mice were allowed to explore freely for 8 min within three closed arms. Performance was evaluated by recording the sequence in which they entered the arms. A sequence where the mouse entered three different arms consecutively was considered one correct alternation. The percentage of spatial working memory was calculated as [number of correct entries/(total number of entries − 2)] × 100%, indicating the mice's spatial memory capacity.

The novel object recognition test was used to assess learning and memory capacities in mice^[24]^. During the training phase, two identical cuboid blocks were placed inside a 50 cm × 50 cm × 45 cm box. Mice were allowed to explore freely for 5 min. After 4 h, one original block was replaced with a novel block of a different shape, and mice were allowed to explore freely for another 5 min. The time spent sniffing both the old and new blocks was recorded to determine preference. The preference percentage was calculated as [sniffing time for the new block/(sniffing time for the old block + sniffing time for the new block)] × 100%.

The sucrose preference test was used to evaluate anhedonia, a symptom of depression in mice^[25–27]^. Mice were adapted to consuming water and a 1% sucrose solution for two days before the formal experiment. Two identical water bottles were placed in a fixed position within a standard rearing cage. On the first day, both bottles were filled with normal drinking water, and the mice were allowed to freely explore the cage for 24 h. On the second day, the bottles were filled with 1% sucrose solution in each test cage. Mice were again allowed to freely explore the cage for 24 h. After acclimation, mice were deprived of water for 12 h, then two weighed bottles (one with 1% sucrose solution and one with water) were placed in each cage. Mice were provided with free access to both bottles for 12 h, and their consumption was measured. Sucrose preference was calculated as [sucrose consumption/(sucrose consumption + water consumption)] × 100%.

### Immunofluorescence (IF) staining

IF staining was performed as previously described^[28]^. Briefly, mice were anesthetized and perfused with 4% paraformaldehyde in phosphate-buffered saline. Then, brains were extracted and fixed in 4% paraformaldehyde for 48 h, dehydrated in 30% sucrose, embedded in Optimal Cutting Temperature compound (Sakura, Torrance, CA, USA), and sectioned into 40-μm thick slices using a freezing microtome (Leica CM 1950; Leica Biosystems, Wetzlar, Hessen, Germany). Sections were stored in a cryoprotective solution containing phosphate-buffered saline (50%), ethylene glycol (30%), and glycerol (20%). Slices were washed and incubated at 4 ℃ for 48 h with the following primary antibodies: anti-BOD1 (1∶1000 dilution; Cat. #HPA036293, Sigma-Aldrich, St. Louis, MO, USA), anti-CaMKⅡα (1∶300 dilution; Cat. #ab22609, Abcam, Cambridge, UK), anti-PV (1∶5000 dilution; Cat. #PV235, Swant, Burgdorf, Switzerland), or anti-c-Fos (1∶1000 dilution; Cat. #226308, Synaptic Systems, Göttingen, Niedersachsen, Germany). Subsequently, the slices were incubated at room temperature for 4 h with the following secondary antibodies: Alexa Fluor 488 anti-mouse IgG (1∶300 dilution; Cat. #A21202, Life Technologies, Carlsbad, CA, USA) and/or Alexa Fluor 594 anti-rabbit IgG (1∶300 dilution; Cat. #A21207, Life Technologies). Finally, images were captured using a confocal microscope (Zeiss LSM 900; Carl Zeiss, Oberkochen, Baden-Württemberg, Germany) and processed by ImageJ software (NIH, Bethesda, MD, USA).

### Stereotaxic injection

Mice were anesthetized with a 2% isoflurane/oxygen mixture. A volume of 200 nL of either pAAV-EF1α-DIO-mCherry-P2A-*Bod1*-3×FLAG-WPRE virus or rAAV-EF1α-DIO-mCherry-WPRE virus (5.89 × 10^12^ viral particles per mL, OBiO Technology; Shanghai, China) was microinfused into the mPFC at a rate of 50 nL/min per side using glass pipettes and a stereotaxic device (RWD Life Science; Shenzhen, Guangdong, China). Stereotactic coordinates relative to bregma were: mPFC (anteroposterior: +2.57 mm; mediolateral: ±0.32 mm; dorsoventral: −2.57 mm). After 21 days post-injection, mice were used for related experiments.

### Brain slice preparation

Three- to four-week-old male mice were anesthetized and sacrificed, and brain tissues were removed and placed in ice-cold artificial cerebrospinal fluid (ACSF; 0.5 mmol/L CaCl_2_, 7 mmol/L MgCl_2_, 87 mmol/L NaCl, 2.5 mmol/L KCl, 75 mmol/L sucrose, 25 mmol/L glucose, 25 mmol/L NaHCO_3_, and 1.25 mmol/L NaH_2_PO_4_) saturated with 5% CO_2_ and 95% O_2_^[29]^. The 300-μm slices of the mPFC were prepared using a vibratome (Leica VT 1200S; Leica Biosystems). Slices were recovered in ACSF at 34 ℃ for 30 min, then at room temperature for one hour before whole-cell patch-clamp recording.

### Whole-cell recordings

Whole-cell recordings of PV^+^ interneurons and pyramidal neurons (PNs) in the mPFC were performed using a MultiClamp 700B amplifier and 1440A digitizer (Molecular Devices, San Jose, CA, USA), as previously reported^[30]^, with glass micropipettes (3.5–5.5 MΩ). PV^+^ interneurons expressing mCherry were visualized with a laser optics microscope equipped with a ×40 water-immersion lens (Olympus, Tokyo, Japan).

The internal solution for intrinsic membrane properties and action potential (AP) frequency recordings contained: 20 mmol/L KCl, 130 mmol/L K-gluconate, 10 mmol/L HEPES, 0.2 mmol/L EGTA, 4 mmol/L Mg-ATP, and 0.5 mmol/L Na_3_-GTP (pH adjusted to 7.30 using KOH). In current-clamp mode, both PV^+^ interneurons and PNs were held at −70 mV, and their membrane properties and AP firing were measured^[31]^. The membrane time constant (*τ*) was fitted by an exponential function of the membrane potential change in response to a rectangular hyperpolarizing current injection, causing a small (approximately 3 to 5 mV) voltage deflection. Input resistance (*R*_in_) was measured using a −60–+10 pA gradient hyperpolarizing current in 10-pA increments and calculated from the slope in the linear phase of current-voltage plots. Membrane capacitance (*C*_m_) was obtained by dividing the τ by the *R*_in_. APs of PV^+^ interneurons were recorded using a depolarizing current of 200–600 pA in 40-pA steps, while APs of PNs were recorded at a 500-ms depolarizing current of 0–300 pA in 20-pA steps.

The internal solution for total K^+^ currents, inward rectifying K^+^ currents, voltage-gated (HCN) K^+^ currents (delayed rectifier), and hyperpolarization-activated cyclic nucleotide-gated currents was identical to the intrapipette solution for AP recording^[32]^. Total K^+^ currents were elicited at −120 mV for 300 ms, followed by −80 mV to +70 mV for 400 ms in 10-mV increments, in the presence of tetrodotoxin (TTX; 1 μmol/L; Tocris, Bristol, UK). Voltage-gated K^+^ channels (delayed rectifier) were recorded at −90 mV to +40 mV for 500 ms in 10-mV increments, in the presence of 4-aminopyridine (4-AP; 2 mmol/L; Tocris) and TTX (1 μmol/L; Tocris). HCN currents were generated from −40 mV to −130 mV for 1000 ms in 10 mV increments.

In voltage-clamp mode, inward rectifying K^+^ currents were recorded at a holding potential of −70 mV. CsCl (1 mmol/L, Sigma-Aldrich) was applied to isolate inward rectifying K^+^ currents, which were subtracted from the current-voltage (I-V) curve recorded from −150 mV to −60 mV in the presence of TTX (1 μmol/L; Tocris). Recordings were conducted with resistance less than 20 MΩ. Data were analyzed using Clampfit 10 (Molecular Devices) and MATLAB (MathWorks, Natick, MA, USA). All drugs and reagents were purchased from Sigma-Aldrich or Tocris.

### Surgery and electrode implantation

Implantations were performed using a rodent stereotaxic instrument. A scalp incision was made, and a small burr hole was drilled into the skull using a dental drill. A stainless steel electrode was then implanted into the left mPFC using a micromanipulator to monitor neuronal activity. After implantation, the mice were returned to their home cages to recover for at least one week.

### *In vivo* electrophysiological data acquisition and analysis

For local field potential (LFP) recordings, the signals were amplified, filtered at 0.1–300 Hz, and sampled at 1 kHz *via* the CereCube recording system. A MATLAB script was used to analyze the LFP signals. To quantify oscillation bands, power was averaged across ranges of theta (3–12 Hz), beta (15–35 Hz), low-gamma (40–60 Hz), and high-gamma (61–95 Hz) bands. Power spectral analysis of oscillations was performed using the multi-taper method in MATLAB, with a window length of two seconds. The time process was divided into one-second periods with 90% overlap. Power was expressed in decibels by computing 10 × lg(output) and then standardized.

### Statistical analysis

Data were analyzed using GraphPad Prism 8 (GraphPad Software, San Diego, CA, USA) and presented as mean ± standard error of the mean. For comparisons between two groups, an unpaired two-sided Student's *t*-test was used. For analyses involving more than two groups, a one-way ANOVA followed by Tukey's post hoc test was generally applied. For multiple groups with two factors, a two-way ANOVA followed by Sidak's post hoc test or Tukey's multiple comparisons test was used for pairwise comparisons. Statistical significance was indicated by *P* < 0.05.

## Results

### *Bod1* deficiency in PV^+^ interneurons induced social novelty deficits and repetitive behaviors in mice

**Figure 1:**
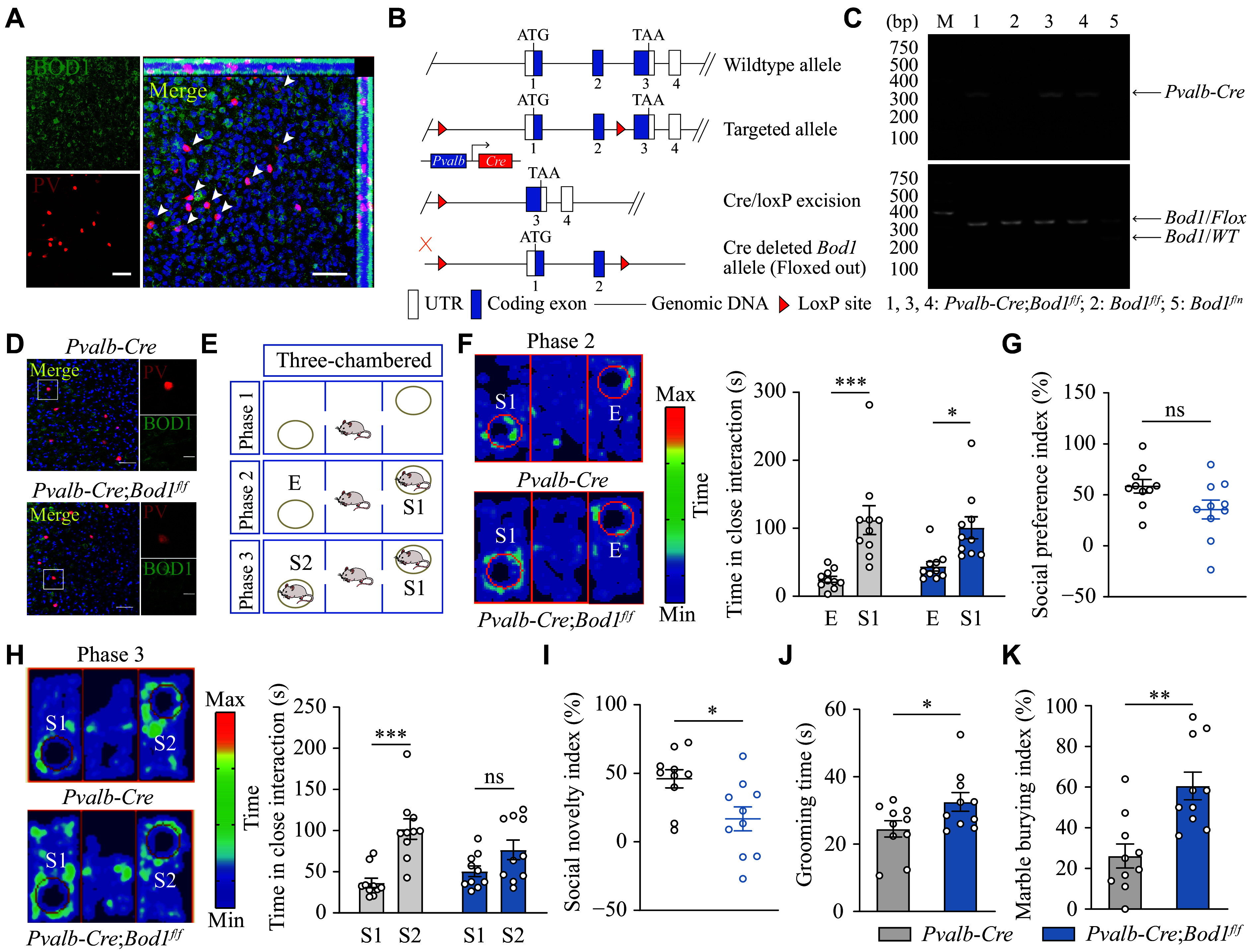
PV^+^ interneuron-*Bod1* deficiency induced social novelty deficits and stereotyped behaviors in mice. A: Representative images of immunofluorescence (IF) staining of BOD1 in PV^+^ interneurons in wild-type mice. BOD1 (green), PV (red), and DAPI (blue; a nuclear marker). Scale bars: 50 μm (left); 20 μm (magnified image, right). Arrowheads indicate BOD1 colocalization in PV^+^ interneurons. The top and right side bars of the enlarged image are the two-dimensional representations derived from Z-stack imaging, demonstrating cellular co-localization patterns; the top bar displays the xz plane and the right-side bar shows the yz plane. B: The strategy for generating PV^+^ interneuron-specific *Bod1* knockout mice (*Pvalb-Cre*;*Bod1*^*f/f*^). Floxed *Bod1* transgenic mice (*Bod1*^*f/f*^) were crossed with *Pvalb-Cre* mice. C: Genotyping of *Pvalb-Cre*;*Bod1*^*f/f*^ mice. *Pvalb-Cre* primers generated a 300-bp product, and *Bod1* primers generated a 300-bp product from the loxP-flanked allele or a 200-bp product from the wild-type allele. D: Representative images of IF staining of BOD1 in PV^+^ interneurons in *Pvalb-Cre* and *Pvalb-Cre*;*Bod1*^*f/f*^ mice. Scale bars: 50 μm (left); 20 μm (zoomed-in image, right). E: Schematic diagram of the three-chamber social test. F: Representative heatmaps of trajectories in phase 2 of the three-chamber social tests (left). The interaction time with the empty cage (E) and the first strange mouse 1 (S1) (right; *n* = 10 per group). G: The social preference index (*n* = 10 per group). H: Representative heatmaps of trajectories in phase 3 of the three-chamber social tests (left). The interaction time with S1 and the new strange mouse 2 (S2) (right; *n* = 10 per group). I: The social novelty index (*n* = 10 per group). J: Quantification of grooming time during the 10-min free movement (*n* = 10 per group). K: The percentage of buried marbles (*n* = 10 per group). Data are presented as mean ± standard error of the mean. ^*^P < 0.05, ^**^P < 0.01, and ^***^P < 0.001. Two-way ANOVA followed by Sidak's post hoc test for F and H; unpaired two-tailed Student's *t*-test for G, I, J, and K. Abbreviations: BOD1, biorientation defective 1; PV, parvalbumin; DAPI, 4',6-diamidino-2-phenylindole; ns, not significant.

We initially examined BOD1 expression in PV^+^ interneurons. IF staining showed that BOD1 colocalized in PV^+^ interneurons (***Fig. 1A***), indicating high expression of BOD1 in these cells. To investigate its role, we generated mice with conditional *Bod1* knockout in PV^+^ interneurons (*Pvalb-Cre*;*Bod1*^*f/f*^; ***Fig. 1B*** and ***1C***). IF staining demonstrated BOD1 loss in PV^+^ interneurons in these mice (***Fig. 1D***). Importantly, PV^+^ interneuron-specific *Bod1* deletion had no effect on body weight, organ index, or brain weight (***Supplementary Fig. 1A***–***1C***, available online).

We then assessed ASD-related behaviors of *Pvalb-Cre;Bod1*^*f/f*^ and *Pvalb-Cre* mice. In three-chamber social interaction tests (***Fig. 1E***), we found that both *Pvalb-Cre;Bod1*^*f/f*^ and *Pvalb-Cre* mice spent more time interacting with the first strange mouse 1 than with the empty cage in the phase 2 test, and there was no significant difference in the social preference index between the groups (***Fig. 1F*** and ***1G***), indicating the normal social ability of *Pvalb-Cre;Bod1*^*f/f*^ mice. However, in the phase 3 test, *Pvalb-Cre;Bod1*^*f/f*^ mice showed no social novelty preference for the new strange mouse 2 and exhibited a lower social novelty index than *Pvalb-Cre* mice (***Fig. 1H*** and ***1I***), indicating the impaired social novelty in *Pvalb-Cre;Bod1*^*f/f*^ mice. Additionally, we found that *Pvalb-Cre;Bod1*^*f/f*^ mice exhibited increased grooming time (***Fig. 1J***) and marble burying index (***Fig. 1K***), compared with *Pvalb-Cre* mice. We further performed Y-maze analysis, novel object recognition, open-field, and sucrose preference tests. The results showed no significant differences in cognition-, motor-, anxiety-, and depression-related behaviors between *Pvalb-Cre;Bod1*^*f/f*^ and *Pvalb-Cre* mice (***Supplementary Fig. 2A***–***2H***, available online). Therefore, these data suggest that *Bod1* deficiency in PV^+^ interneurons may lead to ASD-like behaviors, including social novelty deficits and repetitive behaviors, in mice.

### Hypoactivity of PV^+^ interneurons in the mPFC of *Pvalb-Cre;Bod1*^*f/f*^ mice

**Figure 2:**
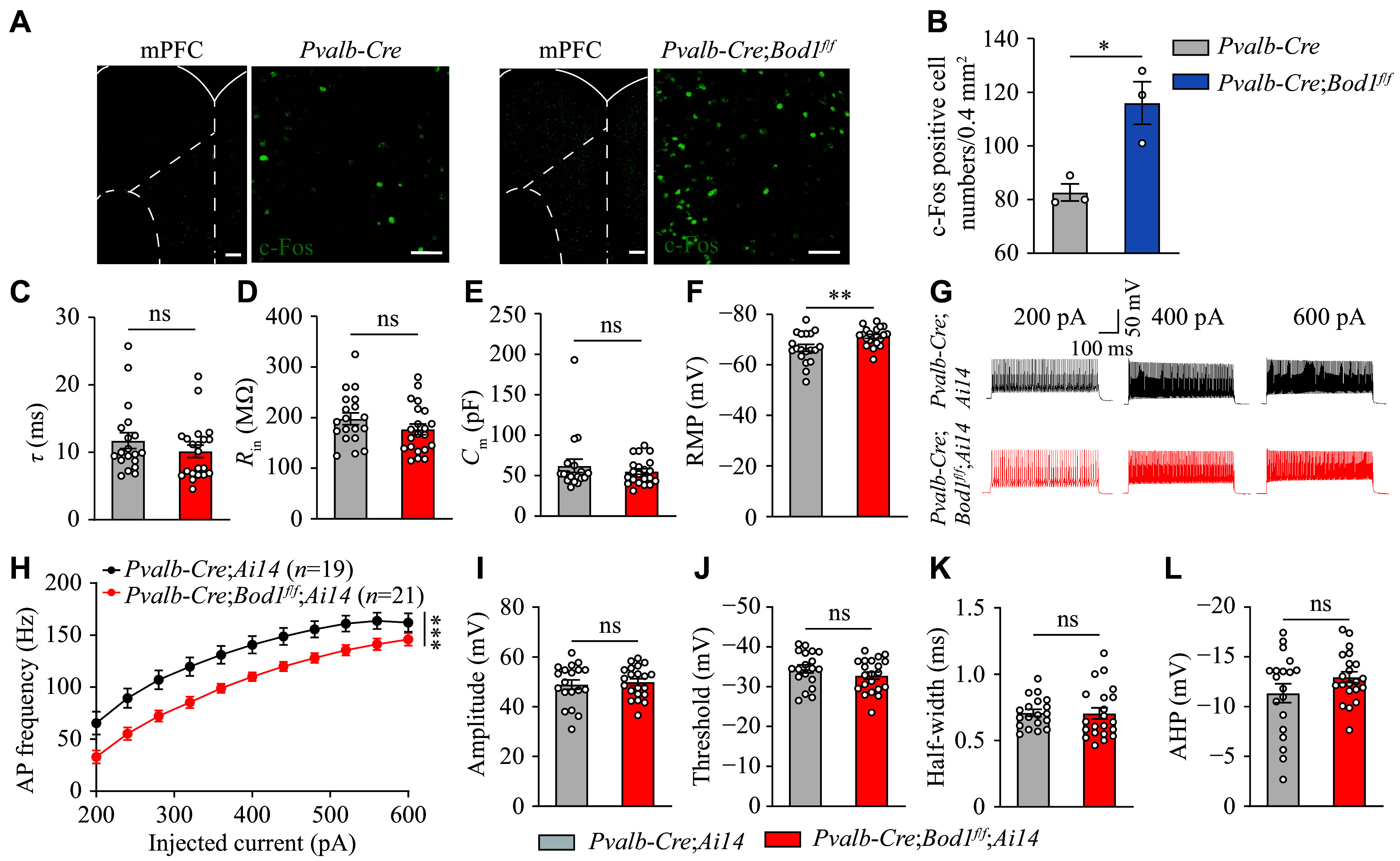
PV^+^ interneurons in the mPFC of *Pvalb-Cre*;*Bod1*^*f/f*^ mice displayed the decreased neuronal excitability. A: Representative confocal images of c-Fos expression following social interaction in the mPFC of *Pvalb-Cre* and *Pvalb-Cre*;*Bod1*^*f/f*^ mice. Scale bar, 50 μm (both the left and right panels). B: Quantitative results of c-Fos-positive cells in the mPFC of *Pvalb-Cre* and *Pvalb-Cre*;*Bod1*^*f/f*^ mice (*n* = 3 per group). C–F: Quantification of membrane properties (*τ*, *R*_in_, *C*_m_, and RMP) in PV^+^ interneurons in the mPFC under whole-cell recording (19 cells from five *Pvalb-Cre;Ai14* mice; 21 cells from five *Pvalb-Cre*;*Bod1*^*f/f*^*;Ai14* mice). G and H: Representative AP firing and quantification of the AP frequency in PV^+^ interneurons in the mPFC of *Pvalb-Cre;Ai14* mice (19 cells from five mice) and *Pvalb-Cre*;*Bod1*^*f/f*^*;Ai14* (21 cells from five mice) following current injections ranging from 200 pA to 600 pA (stepped by 40 pA). I–L: Quantification of the AP properties in PV^+^ interneurons in the mPFC of *Pvalb-Cre;Ai14* mice (19 cells from five mice) and *Pvalb-Cre*;*Bod1*^*f/f*^*;Ai14* (21 cells from five mice). AP amplitude (I), AP firing threshold (J), AP half-width (K), and AP after-hyperpolarization (L). Data are presented as mean ± standard error of the mean. ^*^P < 0.05, ^**^P < 0.01, and ^***^P < 0.001. Unpaired two-tailed Student's *t*-test for B–F and I–L; two-way ANOVA followed by Tukey's multiple comparisons test for H. Abbreviations: mPFC, medial prefrontal cortex; RMP, resting membrane potential; *τ*, time constant; *R*_in_, input resistance; *C*_m_, membrane capacitance; AP, action potential; AHP, after-hyperpolarization potential; ns, not significant.

To identify the key brain region associated with ASD-like behaviors, we examined c-Fos expression, a marker of neuronal activity, in *Pvalb-Cre;Bod1*^*f/f*^ and *Pvalb-Cre* mice following the three-chamber social interaction test. The results showed a significant upregulation of c-Fos expression in the mPFC of *Pvalb-Cre;Bod1*^*f/f*^ mice, compared with *Pvalb-Cre* mice (***Fig. 2A*** and ***2B***), suggesting that mPFC hyperactivity may be linked to ASD-like behaviors in these mice. We also analyzed electrophysiological properties of PV^+^ interneurons in the mPFC through whole-cell patch-clamp recording, and found that the passive membrane properties, as indicated by *τ*, *R*_in_, and *C*_m_, were not significantly altered in the PV^+^ interneurons of the mPFC in *Pvalb-Cre;Bod1*^*f/f*^*;Ai14* mice, compared with those in *Pvalb-Cre;Ai14* mice (***Fig. 2C***–***2E***). However, the resting membrane potential (RMP) was significantly increased (***Fig. 2F***), and the firing rates of AP in PV^+^ interneurons were significantly decreased in *Pvalb-Cre;Bod1*^*f/f*^*;Ai14* mice, compared with *Pvalb-Cre;Ai14* mice (***Fig. 2G*** and ***2H***). Notably, amplitude, threshold, half-width, and after-hyperpolarization potential of AP remained unchanged in *Pvalb-Cre;Bod1*^*f/f*^*;Ai14* mice (***Fig. 2I***–***2L***).

**Figure 3:**
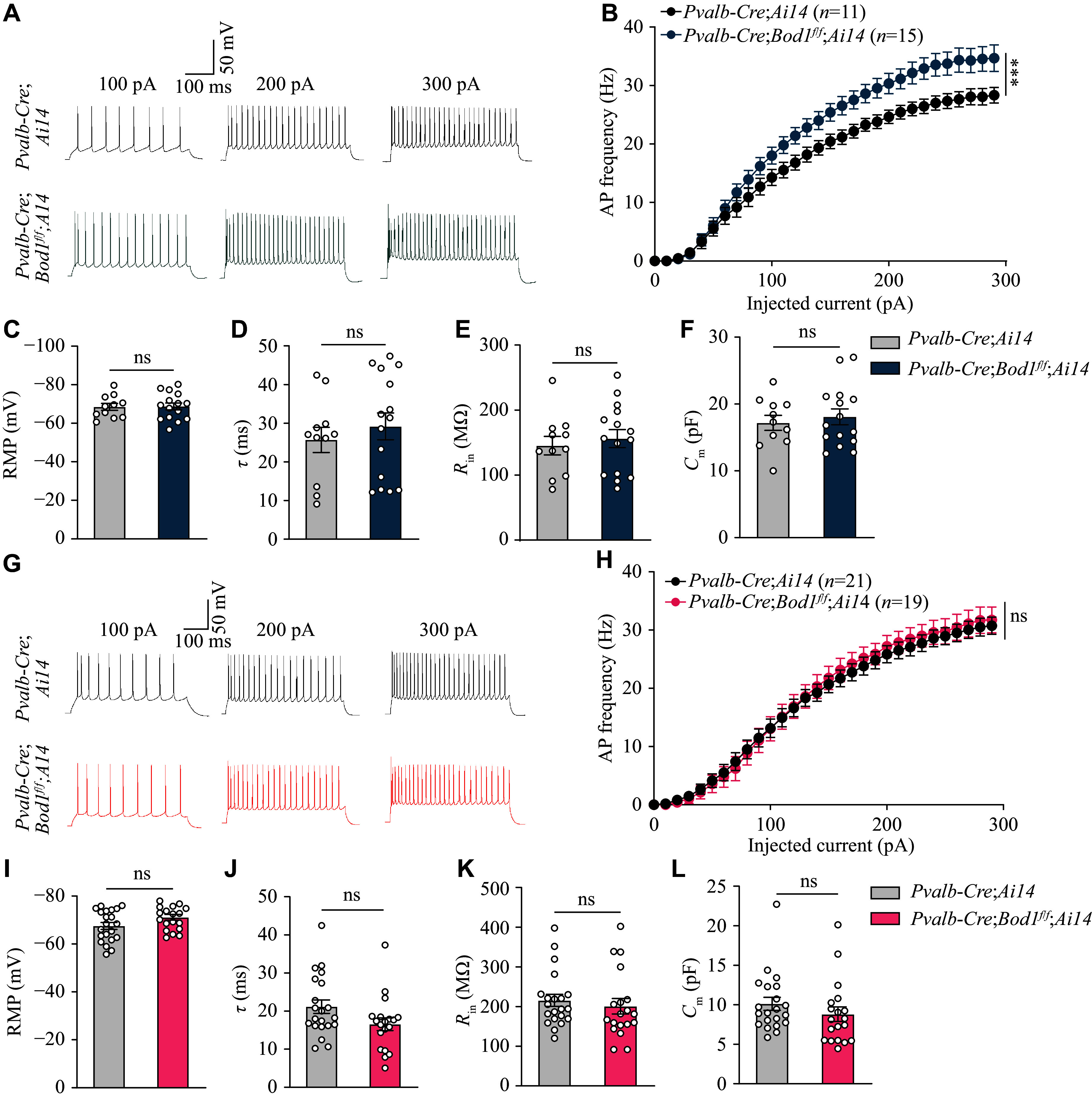
The signature of PNs in the mPFC of *Pvalb-Cre*;*Bod1*^*f/f*^ mice. A and B: Representative traces and quantification of the AP frequency across 0–300-pA current injections (stepped by 20 pA) of mPFC PNs around PV^+^ interneurons (11 cells from three *Pvalb-Cre;Ai14* mice; 15 cells from three *Pvalb-Cre*;*Bod1*^*f/f*^*;Ai14* mice). C–F: Electrophysiological properties (including RMP, *τ*, *R*_in_ and *C*_m_) of PNs around PV^+^ interneurons in the mPFC of *Pvalb-Cre;Ai14* and *Pvalb-Cre*;*Bod1*^*f/f*^*;Ai14* mice (*n* = 3 mice per group). G: Representative traces and quantification of the AP frequency across 0–300-pA current injections (stepped by 20 pA) of mPFC PNs far from PV^+^ interneurons (21 cells from five *Pvalb-Cre;Ai14* mice; 19 cells from five *Pvalb-Cre*;*Bod1*^*f/f*^*;Ai14* mice). I–L: Electrophysiological properties of PNs far from PV^+^ interneurons in the mPFC of *Pvalb-Cre;Ai14* and *Pvalb-Cre*;*Bod1*^*f/f*^*;Ai14* mice (*n* = 5 mice per group). Data are presented as mean ± standard error of the mean. ^***^P < 0.001. Two-way ANOVA followed by Tukey's multiple comparisons test for B and H; unpaired two-tailed Student's *t*-test for C–F and I–L. Abbreviations: RMP, resting membrane potential; *τ*, time constant; *R*_in_, input resistance; *C*_m_, membrane capacitance; ns, not significant.

To further investigate whether *Bod1* deficiency in PV^+^ interneurons affects PN activation, we recorded the firing rates of AP in PNs around or far from the PV^+^ interneurons in *Pvalb-Cre;Ai14* and *Pvalb-Cre;Bod1*^*f/f*^*;Ai14* mice. Under current-clamp conditions, we observed that the firing rates of AP were significantly increased in some PNs around PV^+^ interneurons in *Pvalb-Cre;Bod1*^*f/f*^*;Ai14* mice compared with *Pvalb-Cre;Ai14* mice (***Fig. 3A*** and ***3B***), but showed no significant change in PNs far from the PV^+^ interneurons between the two groups of mice (***Fig. 3G*** and ***3H***). Additionally, the passive membrane properties of all PNs showed no significant differences between the groups (***Fig. 3C***–***3F*** and ***Fig. 3I***–***3L***). These data suggest the microcircuit involving projections from PV^+^ interneurons to PNs in the mPFC.

In summary, our findings indicate that *Bod1* deficiency in PV^+^ interneurons leads to the hypoactivity of PV^+^ interneurons and the hyperactivity of PNs. However, the precise relationship between these neuronal activity changes and ASD-like behaviors remains unclear and warrants further investigation.

### Abnormal gamma oscillations in the mPFC of *Pvalb-Cre;Bod1*^*f/f*^ mice

**Figure 4:**
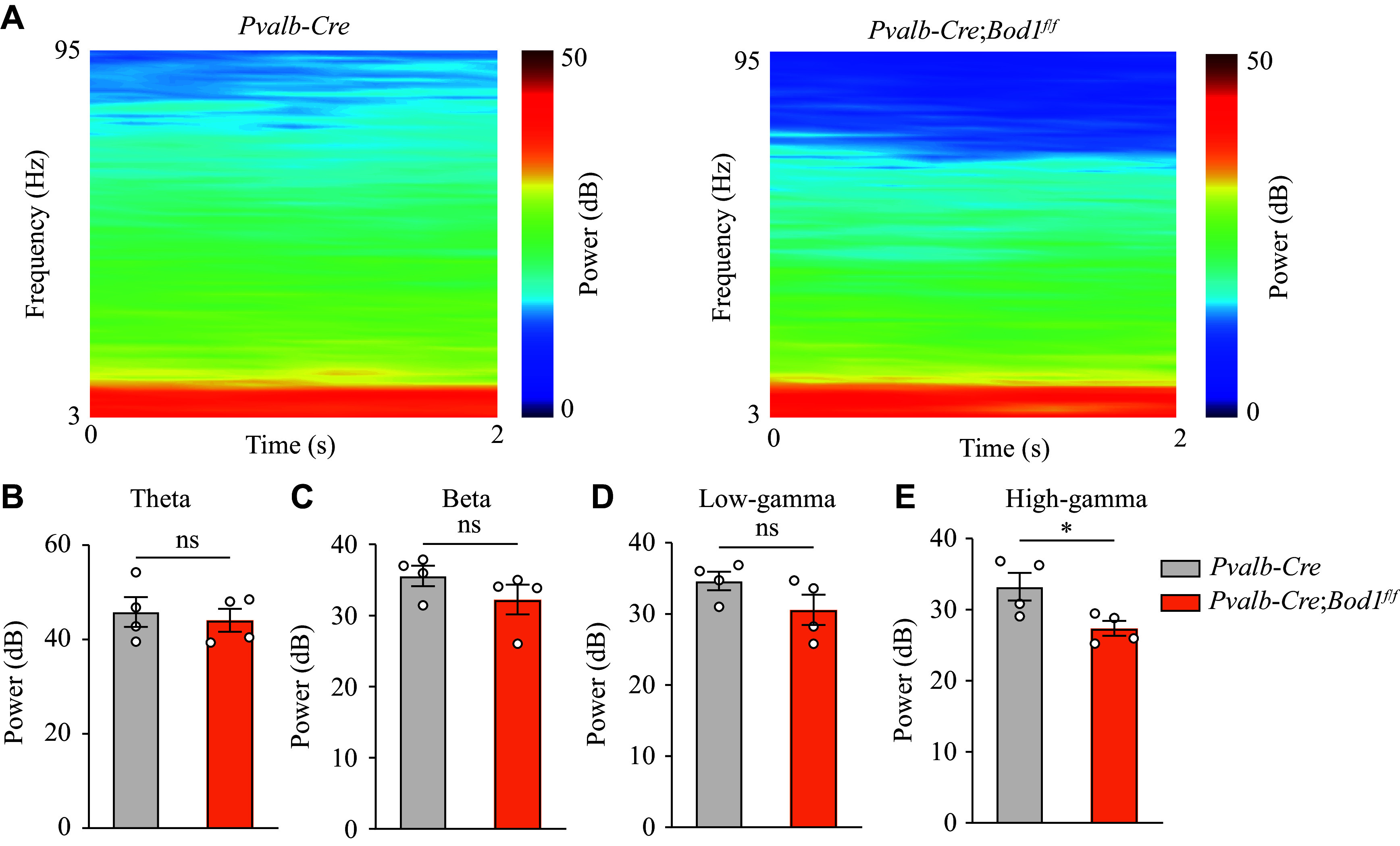
Abnormal gamma oscillations in the mPFC of PV^+^ interneuron-specific *Bod1*-deficient mice. A: Representative spectral power of LFP in the mPFC of *Pvalb-Cre* mice and *Pvalb-Cre*;*Bod1*^*f/f*^ mice. B–E: Relative LFP power values in different frequency bands in the mPFC of mice (*n* = 4 per group). Theta (3 to 12 Hz; C), beta (15 to 35 Hz; D), low-gamma (40 to 60 Hz; E), and high-gamma (61 to 95 Hz; B) power in the mPFC. Data are presented as mean ± standard error of the mean. ^*^P < 0.05. Unpaired two-tailed Student's *t*-test for B–E. Abbreviation: ns, not significant.

Neural oscillation dysfunction in the mPFC has been reported to be involved in autistic behavior^[32]^. To examine the effects of PV^+^ interneuron hypoactivity and PN hyperactivity on synchronized neural oscillations, we implanted multi-channel tetrodes in the mPFC of *Pvalb-Cre* or *Pvalb-Cre;Bod1*^*f/f*^ mice to record the LFPs. Spectrum and power spectral density analyses revealed that *Bod1* deficiency in PV^+^ interneurons resulted in attenuated high-gamma band activity in the mPFC (***Fig. 4A*** and ***4E***), consistent with previous findings that gamma oscillation dysfunction contributes to autistic behaviors^[32]^. However, no significant changes were observed in theta, beta, or low gamma bands (***Fig. 4A***–***4D***). These findings suggest that BOD1 plays an important role in regulating neuron population dynamics.

### Elevation of inward rectifier K^+^ current in PV^+^ interneurons of the mPFC in *Pvalb-Cre;Bod1*^*f/f*^ mice

**Figure 5:**
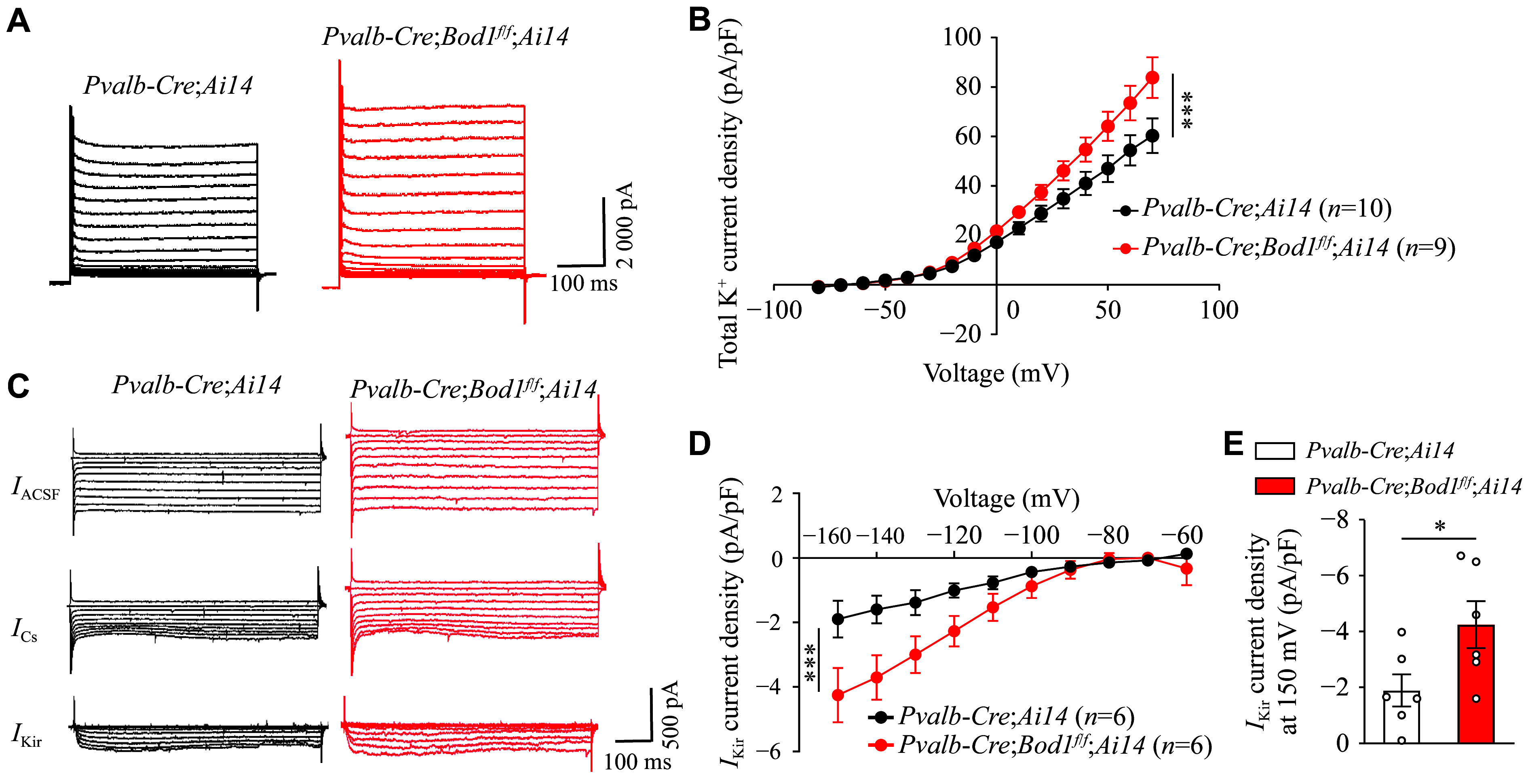
Increased inward rectifying potassium currents in PV^+^ interneurons of the mPFC in *Pvalb-Cre*;*Bod1*^*f/f*^ mice. A and B: Representative K^+^ currents and corresponding summary data for PV^+^ interneurons of the mPFC in *Pvalb-Cre;Ai14* mice (10 cells from three mice) and *Pvalb-Cre*;*Bod1*^*f/f*^*;Ai14* mice (nine cells from three mice). C: Representative traces before (*I*_ACSF_) and after (*I*_Cs_) Cs^+^ perfusion (1 mmol/L CsCl) under voltage steps (–150 mV to –60 mV, stepped by 10 mV, 500 ms duration, holding at –70 mV). Subtraction of the two led to inward rectifying K^+^ current (*I*_Kir_) in *Pvalb-Cre;Ai14* and *Pvalb-Cre*;*Bod1*^*f/f*^*;Ai14* mice. D: The current density of *I*_Kir_ recorded in mPFC PV^+^ interneurons from *Pvalb-Cre;Ai14* mice (six cells from three mice) and *Pvalb-Cre*;*Bod1*^*f/f*^*;Ai14* mice (six cells from three mice). E: Bar graph of current density of *I*_kir_ recorded when neurons were held at −150 mV. Data are presented as mean ± standard error of the mean. ^*^P < 0.05 and ^***^P < 0.001. Two-way ANOVA followed by Tukey's multiple comparisons test for B and D; unpaired two-tailed Student's *t*-test for E. Abbreviations: ACSF, artificial cerebrospinal fluid; Cs, cesium; Kir, inwardly rectifying potassium.

K^+^ channels play important roles in the establishment and maintenance of RMP^[33–34]^. To determine whether *Bod1* deficiency alters K^+^ channel function, we recorded K^+^ currents under voltage-clamp conditions. We found a significant increase in total K^+^ current density in PV^+^ interneurons of the mPFC in *Pvalb-Cre;Bod1*^*f/f*^*;Ai14* mice, compared with *Pvalb-Cre;Ai14* mice (***Fig. 5A*** and ***5B***). Further analysis of specific K^+^ currents, including inward rectifier K^+^ current (*I*_kir_), delayed rectifier K^+^ current (*I*_k_), and hyperpolarization-activated cyclic nucleotide-gated current (*I*_h_), showed no significant changes in *I*_k_ and *I*_h_ densities between the groups (***Supplementary Fig. 3A***–***3E***, available online). However, upon CsCl application to isolate *I*_kir_ currents, we found a significant increase in *I*_kir_ current density in PV^+^ interneurons of the mPFC in *Pvalb-Cre;Bod1*^*f/f*^*;Ai14* mice, compared with *Pvalb-Cre;Ai14* mice (***Fig. 5C***–***5E***). This finding is consistent with previous reports demonstrating that an increased *I*_kir_ contributes to an increased RMP^[35]^. Collectively, these results indicate that *Bod1* deficiency enhances inward rectifier K^+^ currents, leading to a more negative RMP and consequently contributing to the hypoactivity of PV^+^ interneurons in the mPFC.

### BOD1 overexpression in PV^+^ interneurons of the mPFC ameliorated ASD-like behaviors of *Pvalb-Cre;Bod1*^*f/f*^ mice

**Figure 6:**
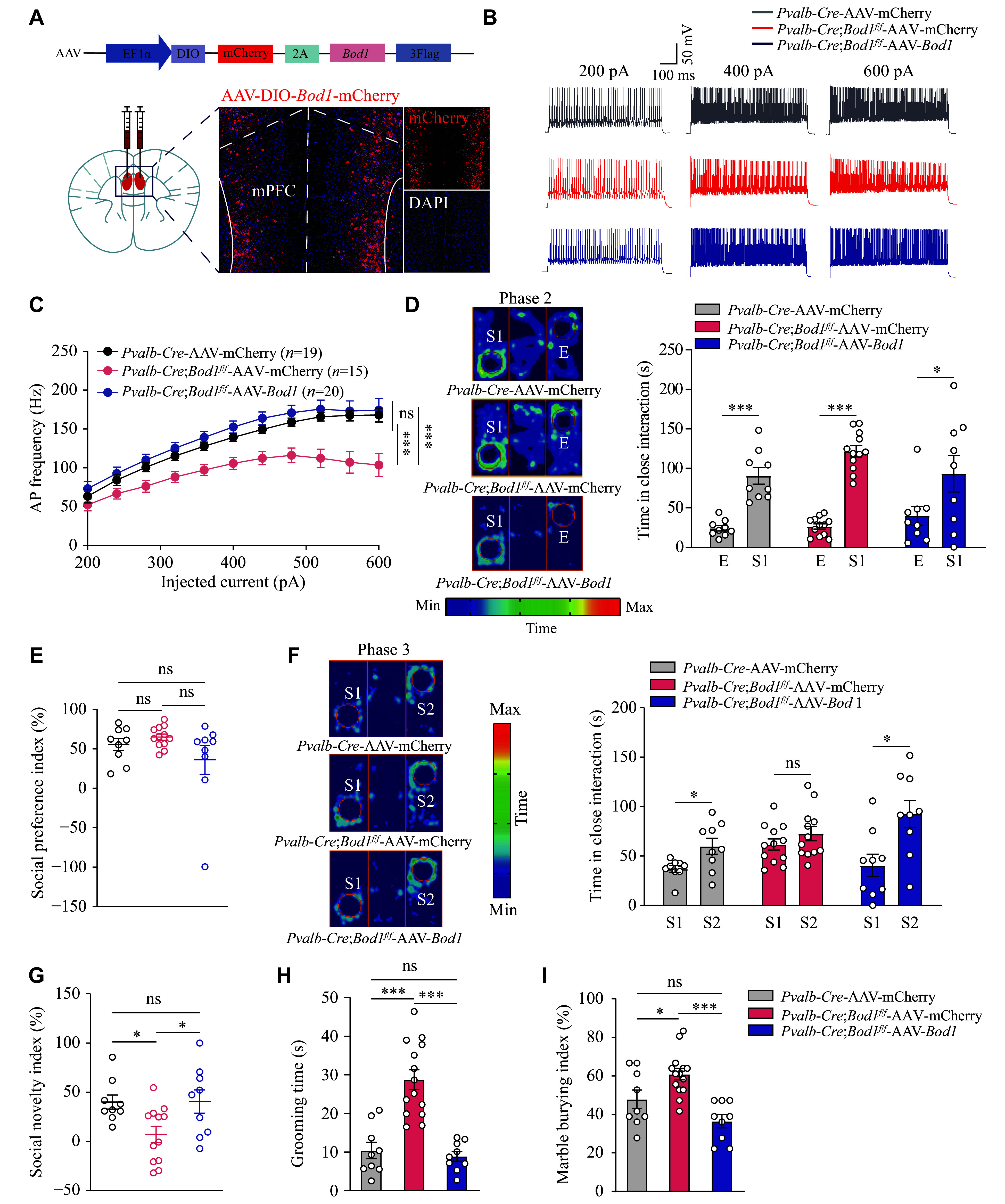
Restoration of BOD1 in mPFC PV^+^ interneurons improved autism-like behaviors in *Pvalb-Cre*;*Bod1*^*f/f*^ mice. A: Schematic of pAAV-EF1α-DIO-mCherry-P2A-*Bod1*-3×FLAG-WPRE (AAV-*Bod1*) constructs and representative images of mCherry (red) after AAV-*Bod1* or control AAV (rAAV-EF1a-DIO-mCherry-WPRE) injection into the mPFC. B: Representative action potential (AP) firing in PV^+^ interneurons of the mPFC. C: Quantification of the AP frequency during current injections ranging from 200–600 pA (stepped by 40 pA) (19 cells from five *Pvalb-Cre*-AAV-mCherry mice; 15 cells from five *Pvalb-Cre*;*Bod1*^*f/f*^-AAV-mCherry mice; 20 cells from five *Pvalb-Cre*;*Bod1*^*f/f*^-AAV-*Bod1* mice). D–I: Quantification of the interaction time (D) and sociability preference index (E) with the empty cage (E) and the strange mouse 1 (S1) in phase 2 of three-chamber tests; time spent interacting (F) and social novelty index (G) with the new strange mouse 2 (S2) in phase 3; the grooming time (H); and the percentage of buried marbles (I) after AAV-mCherry or AAV-*Bod1* injection into the mPFC (*Pvalb-Cre*-AAV-mCherry mice, *n* = 9; *Pvalb-Cre*;*Bod1*^*f/f*^-AAV-mCherry mice, *n* = 12 for D–G and *n* = 14 for H–I; *Pvalb-Cre*;*Bod1*^*f/f*^-AAV-*Bod1* mice, *n* = 9). Data are presented as mean ± standard error of the mean. ^*^P < 0.05 and ^***^P < 0.001. Two-way ANOVA followed by Tukey's multiple comparisons test for C; two-way ANOVA followed by Sidak's post hoc test for D and F; one-way ANOVA followed by Tukey's multiple comparisons test for E and G–I. Abbreviation: ns, not significant.

To further determine whether BOD1 overexpression can alleviate behavioral abnormalities in *Pvalb-Cre;Bod1*^*f/f*^ mice, we injected AAV-DIO-mCherry-*Bod1*-3×Flag (AAV-*Bod1*) or AAV-DIO-mCherry (AAV-mCherry, as control) into the mPFC region of *Pvalb-Cre;Bod1*^*f/f*^ and *Pvalb-Cre* mice. Confocal images showed robust mCherry expression in PV^+^ interneurons within the bilateral mPFC (***Fig. 6A***). Whole-cell patch-clamp recordings revealed that BOD1 overexpression in PV^+^ interneurons effectively restored the decreased AP firing frequency observed in PV^+^ interneurons in *Pvalb-Cre;Bod1*^*f/f*^*;Ai14* mice (***Fig. 6B*** and ***6C***).

Next, we assessed ASD-like behavior. In the three-chamber social interaction tests, all groups spent significantly more time interacting with the first strange mouse 1 than with the empty cage, indicating intact social preference in phase 2 (***Fig. 6D*** and ***6E***). However, in phase 3, the social novelty deficits observed in *Pvalb-Cre;Bod1*^*f/f*^ mice were significantly ameliorated by BOD1 overexpression in PV^+^ interneurons of the mPFC, with the reduced social novelty index fully rescued (***Fig. 6F*** and ***6G***). Moreover, in stereotyped behavior tests, the increased repetitive grooming and increased number of buried marbles in *Pvalb-Cre;Bod1*^*f/f*^ mice were significantly ameliorated by BOD1 overexpression (***Fig. 6H*** and ***6I***).

Taken together, these findings suggest that BOD1 plays a crucial regulatory role in the mPFC in controlling social novelty and stereotyped behaviors. Moreover, BOD1 overexpression in PV^+^ interneurons of the mPFC is sufficient to rescue ASD-like behaviors in *Pvalb-Cre;Bod1*^*f/f*^ mice.

## Discussion

Dysfunction of PV^+^ interneurons has been proposed to be implicated in neurodevelopmental disorders, including ASD^[36–37]^. However, a major challenge remains in elucidating the molecular mechanisms underlying PV^+^ interneuron dysfunctions in ASD. In the present study, we identified several key findings: (1) *Bod1* deficiency disrupts the balance of excitation and inhibition in the mPFC, leading to hypoactivity of PV^+^ interneurons and hyperactivity of PNs; (2) *Bod1* deficiency elevates *I*_Kir_, resulting in a more negative RMP and consequently reducing PV^+^ interneuron excitability; and (3) the gamma oscillation dysfunction in the mPFC, driven by PV^+^ interneuron hypoactivity and PN hyperactivity, may be associated with ASD-like behavioral phenotypes.

PV^+^ interneurons account for approximately 40% to 50% of all interneurons in the mouse cerebral cortex^[38]^. As fast-spiking neurons, they exhibit distinct electrophysiological properties, including a highly depolarized RMP, high-frequency AP firing, small AP amplitudes, and pronounced afterhyperpolarization^[39–40]^. In the present study, we found that *Bod1* deficiency did not alter the passive membrane properties of PV^+^ interneurons, such as *C*_m_, *R*_in_, and *τ*. However, it significantly increased their RMP, leading to a reduction in AP firing frequency. RMP is determined by the balance of ion concentrations across the cell membrane, with high K^+^ conductance playing a key role in stabilizing its negative potential^[33–34]^. K^+^ channels are classified into several families, including voltage-gated K^+^ channels^[41]^, calcium-activated K^+^ channels^[42]^, inwardly rectifying K^+^ (Kir) channels^[43]^, and two-pore domain K^+^ channels^[44]^. Among these, Kir channels are particularly crucial for maintaining the RMP^[35]^. Our findings indicate that *Bod1* deficiency disrupts Kir current function, resulting in the increased RMP. Previous studies have shown that PP2A regulates the surface expression of G protein-gated Kir channels^[45]^, and BOD1 has been reported to modulate PP2A activity^[14,16]^. Based on this, we hypothesize that *Bod1* deficiency may increase Kir current by inhibiting PP2A activity. However, future research is required to validate this hypothesis.

*In vivo* evidence suggests that ASD patients exhibit defects in neural gamma oscillation patterns^[46]^. In the mPFC, gamma oscillation dysfunction has been linked to autistic behavior in neuroligin 3 R451C knockin mice^[12]^. PV^+^ interneurons play a crucial role in generating gamma oscillations in the cerebral cortex^[47]^. Their synchronous activity amplifies gamma oscillations, whereas the reduced activity or inhibition suppresses them^[47–48]^. Consistent with this, our findings demonstrate that *Bod1* loss in PV^+^ interneurons leads to their hypoactivity and results in PN hyperactivity, ultimately causing the reduced high-gamma power and social dysfunction.

Overall, the present study reveals a novel mechanism by which BOD1 regulates Kir channel-mediated currents in PV^+^ interneurons. The loss of BOD1 function in PV^+^ interneurons triggers ASD-like behaviors, while restoring BOD1 function rescues these behaviors. These findings highlight some potential therapeutic targets for neurological disorders associated with PV^+^ interneuron dysfunction.

## Fundings

This work was supported by the National Key Research and Development Program of China (Grant No. 2022YFE0108600 to Y. M. L.) and the National Natural Science Foundations of China (Grant Nos. 82473918 and 82104162 to X. X. L.).

## SUPPLEMENTARY DATA

Supplementary data to this article can be found online.
