# Supplementary material for: Upregulated inwardly rectifying K+ current-mediated hypoactivity of parvalbumin interneurons underlies autism-like deficits in Bod1-deficient mice
Source: J Biomed Res. 2025 Mar 31;39(4):417–29. doi: 10.7555/JBR.38.20240394 (PMC12329414; doi:10.7555/JBR.38.20240394)
Supplement: Supplementary file 1 — Supplementary data to this article can be found online. [file jbr-39-4-417-Supplementary.pdf]

Available online at www.jbr-pub.org.cn

Open Access at PubMed Central

Journal of Biomedical Research, 2025 39(4): 1–2

Supplementary Data

JBR

Upregulated inwardly rectifying K+ current-mediated hypoactivity of parvalbumin interneurons underlies autism-like deficits in *Bod1*-deficient mice

Chen Li[1,△], Kerui Wang[1,△], Xingfeng Mao[2], Takuya Sasaki[3], Xiuxiu Liu[4,✉], Yingmei Lu[1,✉]

[1]Key Laboratory of Modern Toxicology of Ministry of Education; School of Basic Medical Sciences, Nanjing Medical University, Nanjing, Jiangsu 211166, China;
[2]School of Basic Medical Sciences, Nanjing Medical University, Nanjing, Jiangsu 211166, China;
[3]Department of Pharmacology, Graduate School of Pharmaceutical Sciences, Tohoku University, Sendai 980-8578, Japan;
[4]Medical Basic Research Innovation Center for Cardiovascular and Cerebrovascular Diseases, Ministry of Education; International Joint Laboratory for Drug Target of Critical Illnesses; Key Laboratory of Cardiovascular & Cerebrovascular Medicine; School of Pharmacy, Nanjing Medical University, Nanjing, Jiangsu 211166, China.

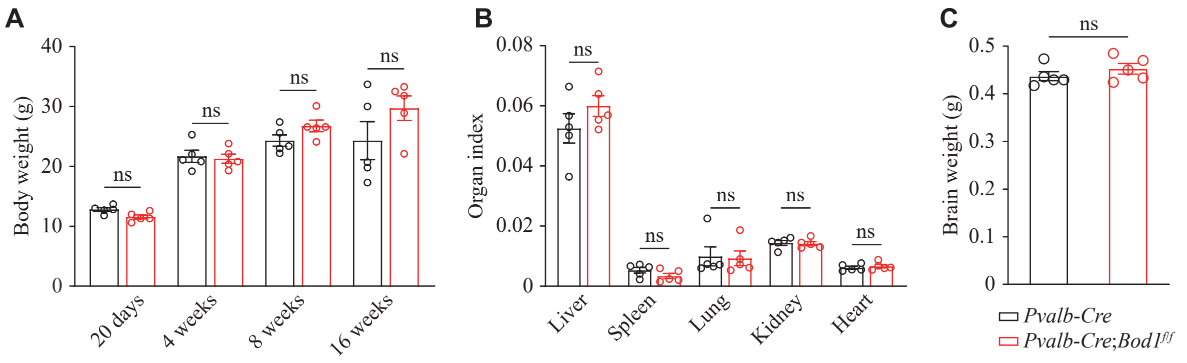

Supplementary Fig. 1 ***Bod1*** **deficiency did not influence body weight or organ weight.** A: Body weight of *Pvalb-Cre* and *Pvalb-Cre*;*Bod1*$^{f/f}$ mice at different ages (n = 5 mice at 20 days, 4 weeks, 8 weeks, and 16 weeks per group). B: Organ-to-body weight ratios of *Pvalb-Cre* and *Pvalb-Cre*;*Bod1*$^{f/f}$ mice (n = 5 mice at 4-week-old per group). C: Brain weight of 4-week-old *Pvalb-Cre* and *Pvalb-Cre*;*Bod1*$^{f/f}$ mice (n = 5 mice at 4-week-old per group). Data are presented as mean ± standard error of the mean. Two-way ANOVA followed by Bonferroni's post hoc test for A and B; unpaired two-tailed Student's t-test for C. Abbreviations: ns, not significant.

△ These authors contributed equally to this work.

✉Corresponding authors: Xiuxiu Liu, Medical Basic Research Innovation Center for Cardiovascular and Cerebrovascular Diseases, Ministry of Education; International Joint Laboratory for Drug Target of Critical Illnesses; Key Laboratory of Cardiovascular & Cerebrovascular Medicine; School of Pharmacy, Nanjing Medical University, 101 Longmian Avenue, Nanjing, Jiangsu 211166, China. E-mail: xiuxiuliu@njmu.edu.cn; Yingmei Lu, Key Laboratory of Modern Toxicology of Ministry of Education; School of Basic Medical Sciences, Nanjing Medical University, 101 Longmian Avenue, Nanjing, Jiangsu 211166, China. E-mail: lufx@njmu.edu.cn.

Received: 14 November 2024; Revised: 05 March 2025; Accepted: 14 March 2025; Published online: 31 March 2025

CLC number: R749.94, Document code: A

The authors reported no conflict of interests.

This is an open access article under the Creative Commons Attribution (CC BY 4.0) license, which permits others to distribute, remix, adapt and build upon this work, for commercial use, provided the original work is properly cited.

© 2025 by Journal of Biomedical Research.

https://doi.org/10.7555/JBR.38.20240394

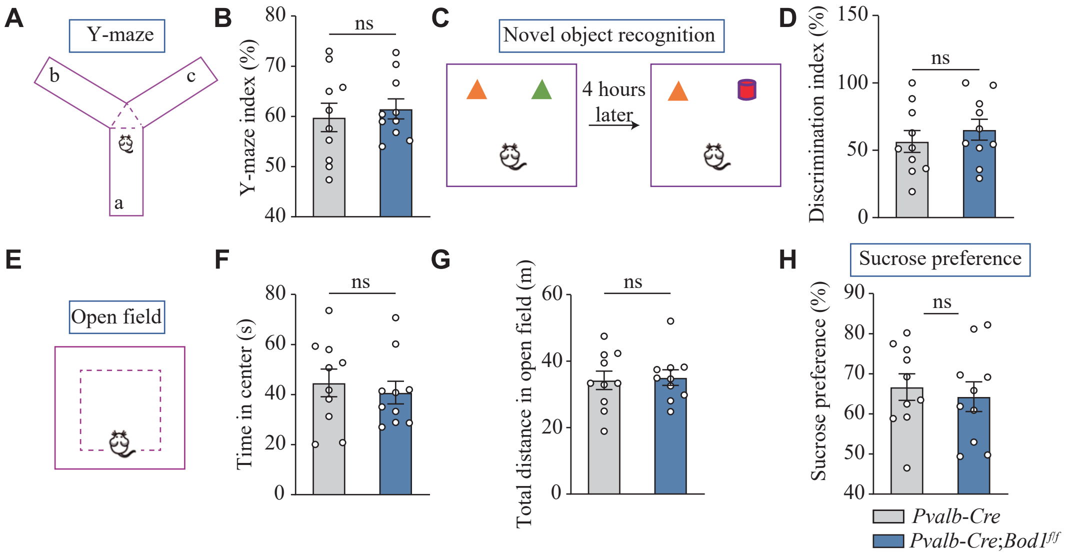

Supplementary Fig. 2 ***Pvalb-Cre*;*Bod1$^{f/f}$*** **mice did not exhibit learning or memory deficit, motor ability disorder, anxiety-like behaviors, or depression-like behaviors.** A: Behavior paradigms of Y-maze test. B: Quantification of spontaneous alternation in Y-maze test. C: Schematic of the novelty recognition test. D: The discrimination index in the novel object recognition test. E: Diagram of the open field test. F: Quantitation of the time in center zone. G: Quantification of total distance in the 5-min open field test. H: The sucrose preference index in the sucrose preference test. n = 10 for each group. Data are presented as mean ± standard error of the mean. Unpaired two-tailed Student's t-test for B, D, and F–H. Abbreviations: ns, not significant.

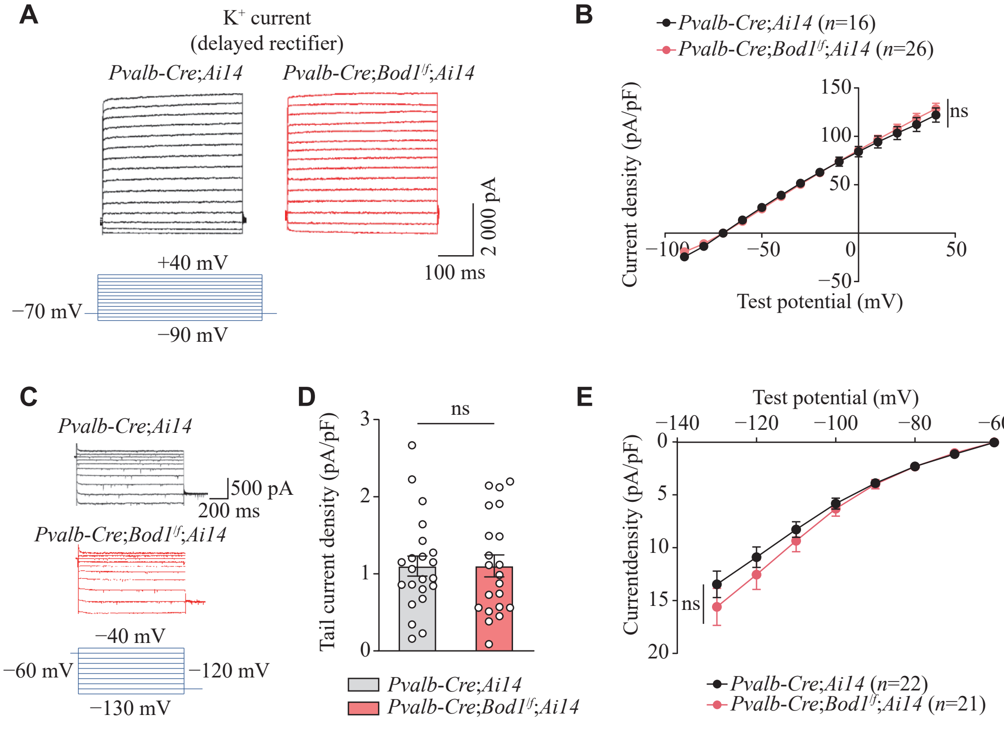

Supplementary Fig. 3 ***Pvalb-Cre*;*Bod1$^{f/f}$*** **mice displayed no significant alterations in delayed rectifier K^+ currents and I_h currents.** A: Representative delayed rectifier K^+ current traces of mPFC PV^+ interneurons under whole-cell voltage-clamp recordings. B: The summary data of delayed rectifier K^+ currents recording in PV^+ interneurons of the mPFC (16 cells from five *Pvalb-Cre;Ai14* mice; 26 cells from five *Pvalb-Cre*;*Bod1$^{f/f}$;Ai14* mice). C: Representative I_h current traces in PV^+ interneurons of the mPFC from a whole-cell voltage-clamp recording. D: Summary of I_h tail current recordings in *Pvalb-Cre;Ai14* and *Pvalb-Cre*;*Bod1$^{f/f}$;Ai14* mice (22 cells from five *Pvalb-Cre;Ai14* mice; 21 cells from five *Pvalb-Cre*;*Bod1$^{f/f}$;Ai14* mice). E: The I_h current density recorded under hyperpolarizing steps at indicated voltages in PV^+ interneurons of the mPFC from *Pvalb-Cre;Ai14* mice (22 cells from five mice) and *Pvalb-Cre*;*Bod1$^{f/f}$;Ai14* mice (21 cells from five mice). Data are presented as mean ± standard error of the mean. Two-way ANOVA followed by Tukey's multiple comparisons test for B and E; unpaired two-tailed Student's t-test for D. Abbreviations: ns, not significant.
